# Leveraging genomic prediction to surpass current yield gains in spring barley

**DOI:** 10.1007/s00122-024-04763-1

**Published:** 2024-11-06

**Authors:** Johanna Åstrand, Firuz Odilbekov, Ramesh Vetukuri, Alf Ceplitis, Aakash Chawade

**Affiliations:** 1 Department of Plant Breeding, Swedish University of Agricultural Sciences, Alnarp, Sweden; 2 Lantmännen Lantbruk, Svalöv, Sweden

## Abstract

**Key message:**

Genetic gain in Nordic spring barley varieties was estimated to 1.07% per year. Additionally, genomic predictive ability for yield was 0.61 in a population of breeding lines.

**Abstract:**

Barley is one of the most important crops in Europe and meeting the growing demand for food and feed requires continuous increase in yield. Genomic prediction (GP) has the potential to be a cost-efficient tool in breeding for complex traits; however, the rate of yield improvement in current barley varieties is unknown. This study therefore investigated historical and current genetic gains in spring barley and how accounting for row-type population stratification in a breeding population influences GP results. The genetic gain in yield was estimated using historical data from field trials from 2014 to 2022, with 22–60 market varieties grown yearly. The genetic gain was estimated to 1.07% per year for all varieties, serving as a reference point for future breeding progress. To analyse the potential of using GP in spring barley a population of 375 breeding lines of two-row and six-row barley were tested in multi-environment trials in 2019–2022. The genetic diversity of the row-types was examined and used as a factor in the predictions, and the potential to predict untested locations using yield data from other locations was explored. This resulted in an overall predictive ability of 0.61 for yield (kg/ha), with 0.57 and 0.19 for the separate two-row and the six-row breeding lines, respectively. Together this displays the potential of implementing GP in breeding programs and the genetic gain in spring barley market varieties developed through GP will help in quantifying the benefit of GP over conventional breeding in the future.

**Supplementary Information:**

The online version contains supplementary material available at 10.1007/s00122-024-04763-1.

## Introduction

Barley (*Hordeum vulgare*) is the third most important crop in Europe with 91 million tons produced in 2022 (FAO-STAT 2024). While mainly used for feed and malting, the nutritious cereal is gaining popularity for human consumption (Baik and Ullrich 2008; Meints et al. 2016). Breeding for higher sustainable yields in a changing climate and introducing new varieties into new locations is vital to meet the growing demand for food stability. The success of breeding programs has been measured by the level of adoption of the newly released varieties by the growers (Brennan and Byerlee 1991). Another measurement is the rate of genetic gain in varieties on the market (Rutkoski 2019). Genetic gain, or genetic response, is a measure of the improvement in average genetic or phenotypic value in a population due to repeated artificial selection (Hazel and Lush 1942). The genetic gain per unit time is estimated through the breeder’s equation $R=\frac{i{\sigma }_{A}r}{t}$ and is dependent on the selection intensity (*i*), the square root of the additive genetic variance (${\sigma }_{a}$), the selection accuracy (*r*) which is the narrow sense heritability (*h*^*2*^) in phenotypic selection, and the cycle time (*t*). Several studies have investigated the genetic gain of barley in breeding programs around the world from the late 1800s and onwards, summarized in Table 1 and reviewed in Cossani et al. (2022). These have generally found advancement in genetic gain of 14.9–74 kg/ha per year or 0.4–1.1% per year; however, the studies often involve very few samples, sometimes with single varieties representing each decade of breeding (Table 1). There also seems to be a difference in the achieved genetic gain between the row-types in barley where the two-rowed lines have improved more in yield compared to the six-rowed varieties tested (Martiniello et al. 1987; Munoz et al. 1998). In addition, the genetic improvement has not been steady over time, instead the increase in genetic gain for yield was higher in the second half of the 1900s (Riggs et al. 1981; Rodrigues et al. 2020).

**Table 1:** Genetic gain estimates of spring and winter barley in breeding programs in different regions 1880–2013

Country/Region	Type and number of lines	Years	Genetic gain in yield per year	References
Argentina	Spring: 9 two-row	1944–1998	41 kg/ha or 0.36%	Abeledo et al. (2003)
Australia	Spring: 13 two-row	1942–2013	16 kg/ha or 0.43%	Cossani et al. (2022)
Brazil	Spring: 5 two-row 1 per decade	1968–2008	No gain until 1980, then 59.9 kg/ha	Rodrigues et al. (2020)
Canada	Spring: 20 six-row	1910–1988	26 kg/ha	Bulman et al. (1993)
Canada	Spring: 20 genotypes	1910–1987	41.1 kg/ha or 0.17% of harvest index	Jedel and Helm (1994)
England and Wales	Spring: 37 genotypes	1880–1980	15 kg/ha (0.39% per year) in 1880–1953, and 41 kg/ha (0.84% per year) in 1953–1980	Riggs et al. (1981)
Finland	Spring: 7 six-row 1 per decade	1927–1980	No gain	Peltonen-Sainio and Karjalainen (1991)
Italy	Winter: 5 two-row, 12 six-row	1960–1980	74 kg/ha or 1.1% (two-row) 52 kg/ha or 0.75% (six-row)	Martiniello et al. (1987)
Nordic Region	Spring: 90 two-row, 29 six-row	1930–1991	22 kg/ha or 0.28%	Ortiz et al. (2002)
Spain	Winter and spring: 10 two-row, 10 six-row	1930–1990	40.7 kg/ha for two-row 32.9 kg/ha for six-row	Munoz et al. (1998)
USA	Spring: 6 genotypes *1 per decade*	1920–1982	45.7 kg/ha or 0.9%	Wych and Rasmusson (1983)
USA	Spring: 10 genotypes	1920–1984	15.7 kg/ha per year	Boukerrou and Rasmusson (1990)
USA	Spring: 98 genotypes	1958–1998	14.9 kg/ha or 0.4%	Condón et al. (2009)

In the Nordic region, the genetic gain has been analyzed in small populations from 1927 to 1980, where no gain was found (Peltonen-Sainio and Karjalainen 1991), and in 119 varieties from 1930 to 1991 with a gain of 22 kg/ha or 0.28% (Ortiz et al. 2002), but to our knowledge there have been no recent studies evaluating current progress in plant breeding. By periodically tracking the genetic gain in the current market varieties, it is possible to provide a reference point that can be used to evaluate new breeding strategies.

The continued improvement of spring barley is needed, which can be aided by incorporating genomic prediction (GP) in breeding programs. GP aims to predict breeding and genetic values based on marker effects (Bernardo 1994; Meuwissen et al. 2001). In GP, the genotypic and phenotypic data of a training population are used to find the genomic estimated breeding values (GEBVs) of individuals in a testing population where there is genetic but not phenotypic data available (Meuwissen et al. 2001). The GEBV is the sum of all marker effects estimated in the model from the accessions with both genotype and phenotype records and can be used to make predictions of the breeding value of non-phenotyped individuals based on their genotypic scores. The accuracy of GEBVs is defined as the Pearson correlation between GEBV and the true breeding value. Breeding programs with incorporated GP methods have the potential to achieve a higher genetic gain in a variety of species compared to breeding based on only phenotypic selection, through the optimization of the parameters in the breeder’s equation, i.e., increased selection accuracy, selection intensity, and cycling time (Crossa et al. 2017). GP as a breeding strategy also led to a higher stability in the genetic gain over time when genotype by year interaction is present (Gaynor et al. 2017).

In barley, GP has been carried out with different combinations of models, marker densities, and population compositions with varying predictive abilities (PAs). Rembe et al. (2022) achieved a PA of 0.73 for grain yield; however, with a leave-one-out strategy of different locations and years the PA of the model decreased to a range of 0.14–0.46. In another study with a low-density SNP dataset of 337 markers and only six-rowed breeding lines, the PA of yield was 0.54 (Tiede and Smith 2018). Thorwarth et al. (2017) found only minor differences between GP models when comparing eleven different GP models for German six-rowed winter barley and suggested instead that the population structure significantly affected the results.

A complicating factor in barley breeding programs is the genetic difference between the two-row and the six-rowed barley lines (Wonneberger et al. 2023); however, studies on GP using combined populations of two-rowed and six-rowed barley are not common. One study on naked barley germplasm used a diversity panel of two-row and six-row barley for the prediction of threshability where the subpopulations were found to differ significantly (Massman et al. 2023). This resulted in an overall PA of 0.86; however, within five out of six separate subpopulation the PA was lower than within the combined population (Massman et al. 2023). Where significant subpopulation structures with differing between-group PA, e.g., where both barley row-types exist within a breeding programe, it is important to consider population effects when designing a training population to avoid an inflated estimate of the PA for the entire population.

In this study, the genetic gain in spring barley varieties was investigated. A set of lines available on the market and prospective new lines were compared against cultivars with current or previously large market shares in Sweden. Historical data from official trials were used to estimate the progress of commercial lines released and registered to the market between 2001 and 2023. Field trial data from a breeding programe of two-row and six-row spring barley were also used to develop GP models. Yield data collected from multi-environment trials were used to develop and test models for predicting yield in specific targeted environments. The influence of the row-type and the breeding set on the predictive ability was investigated.

## Materials and methods

### Genetic gain analysis

Field data were collected from national multi-environment trials of market varieties of two-row spring barley typically grown in Sweden through Sverigeförsöken, conducted by Hushållningssällskapet and the Swedish University of Agricultural Sciences. For 2014–2022, data were collected from four locations in southern Sweden each year, with twelve different trial sites used. In each location the lines were tested in an alpha lattice field design in two–three replicates. Each replicate consisted of five to eight sub-blocks containing four to eight lines depending on the trial site and the number of lines tested. Statistical analysis of the field trials was carried out per trial basis by Sverigeförsöken (https://sverigeforsoken.se/). Trials with three replicates were analyzed by using an incomplete block design with one treatment factor and the replicates as a random effect. Trials with two replicates were analyzed using a split-plot incomplete block analysis with replications with either random or fixed effects. The fields were treated according to local best practices for each location, adding fertilizer, fungicide, and herbicide. The number of lines tested each year varied from 22 to 60, with a total of 174 unique lines that represented common market varieties grown in Sweden, registered from 2001 to 2023, Supplemental Table 1 (Online resource 1). Yield was given as tons/hectare harvested grain at 15% moisture content.

Each year three checks were included: KWS Irina, RGT Planet, and Dragoon. The adjusted yield from the field design analysis of these three checks was averaged within each trial site to form a synthetic tester. To obtain a relative yield of each line, the yield of the tested line was divided by the yield of the synthetic tester within each environment. Genetic gain (GG) over time was estimated in two ways: as a population of varieties and as individual varieties available on the market. For the population of market varieties, the GG was estimated by plotting the relative yield of each line in the year it was tested, and performing regression analysis where the slope of the line over the years was used as the GG estimate. For the estimation of the improvement of released individual market varieties, the relative yield of an accession was averaged in all its tested years to obtain a measurement of its performance across environments. This relative yield was then plotted against the year of registration which was obtained from VarietyFinder (CPVO, www.cpvo.europa.eu) to determine the genetic gain over time using individual lines.

### Plant material and field data for genomic prediction

Spring barley breeding lines from the Lantmännen plant breeding company were tested in three locations in Sweden for three years, 2019–2021. The population comprised 375 spring barley breeding lines with 222 two-row and 153 six-row lines. The lines were developed in two breeding sets with 178 lines in SWA18 (74 two-row and 104 six-row) and 195 (147 two-row and 48 six-row) in SWA19 and included two checks, Anneli (two-row) and SWJudit (six-row). The lines were tested at locations of different latitudes of 63.2 (Location 1), 59.6 (Location 2) and 58.3 (Location 3) to study the performance at different daylight and temperature conditions. All 375 lines were tested in all locations and years and were planted in 15 m^2^ plots in a non-replicated trial consisting of 42 blocks. Each block contained 39 entries in each block of which nine were checks; either Anneli or SWJudit was used in each block. The fields were treated according to local best practices with fertilizer and herbicide in all locations and fungicide in location 2 and 3. Yield was given per plot as kg/hectare at a target water moisture content of 15%.

### Phenotype analysis

To control for differences in field conditions, each yield plot was normalized to a nearby check to estimate the adjusted mean yield of each genotype. This was carried out by using the linear methods of moving means analysis (Townley-Smith and Turd, 1973) in Genovix (Agronomix Software 2022) to obtain yield values adjusted to local checks within each environment. The adjusted means of each line were used to calculate the best linear unbiased predictor (BLUP) values using the lme4 package version 1.1–35.1 implemented in R 4.2.2 (Bates et al. 2015) with genotype, location, and year set as random effects. The following formula module was used to estimate BLUPs for each line in the population:

1 $${\text{BLUP}} = yield\sim \left( {1|gen} \right) + \left( {1|loc} \right) + \left( {1|year} \right) + \left( {1|gen:loc} \right) + \left( {1|gen:year} \right) + \left( {1|loc:year} \right) + \left( {1|loc:year:block} \right)$$

where yield is the adjusted mean yield in kg/ha, gen is the genotype, loc is the trial location (Location 1–3), year is the trial year (2019–2021), gen:loc is the genotype and location interaction, gen:year is the genotype and year interation, loc:year is the interaction between the location and the year, and loc:year:block is the interaction between the location, year and the block effect of the non-replicated trial design. For downstream prediction evaluations within subgroups the BLUP values were calculated with lines unique to the subgroup. The lines belonging to different row-types and breeding sets were subset prior to the separate BLUP value estimation. For single environment predictions all locations and years for all lines were used for the BLUP estimation except for the environment to be predicted, i.e., two years with three locations and one year with two locations. BLUP values for individual locations and years were calculated using the entire population but with the following adjustments of the Eq. 1 for year and location. Individual years were calculated as:

2 $${\text{BLUP }} = \, yield \, \sim \, \left( {1|gen} \right) + \left( {1|loc} \right) + \left( {1|gen:loc} \right) + \left( {1|loc:block} \right)$$

3 $${\text{BLUP }} = yield \, \sim \, \left( {1|gen} \right) \, + \, \left( {1|year} \right) \, + \, \left( {1|gen:year} \right) \, + \, \left( {1|year:block} \right)$$

Broad sense heritability was calculated according to:

4 $$H^{2} = \frac{{\sigma_{g}^{2} }}{{\sigma_{g}^{2} + \frac{{\sigma_{gy}^{2} }}{{n_{y} }} + \frac{{\sigma_{gl}^{2} }}{{n_{l} }} + \frac{{\sigma_{r}^{2} }}{{n_{y} n_{l} }}}}$$

where ${\sigma }_{g}^{2}$, ${\sigma }_{gy}^{2}$, ${\sigma }_{gl}^{2}$, and ${\sigma }_{r}^{2}$ are the variance components of genotype, genotype × year, genotype × location, and residual variance, respectively, and the ${n}_{y}$, and ${n}_{l}$ are the number of years, and locations per environment (Schmidt et al. 2019). The yield in each environment was plotted against the BLUP values estimated from all environments using ggplot2 version 3.4.4 (Wickham 2016) to visualize the relationship between yield in single environments and the adjusted values.

### Genetic analysis of the population

The population was genotyped with the Illumina Infinium XT 15k SNP array by TraitGenetics (SGS Institut Fresenius, Germany), with 13,847 SNP markers. The markers were filtered by removing markers with more than 10% missing values in the entire population and markers without an annotated position in the barley genome, leaving 12,559 markers. A total of 9589 markers remained when filtering the entire population for a minor allele frequency (MAF) of > 0.05, whereas 7351 and 5624 markers remained when filtering two-row (221 lines) and six-row (152 lines) separately. When filtering for MAF above 0.05 in the entire population 4.6% of the polymorphic markers within each row-type were removed, 1.5% and 3.1% from the two-rowed and six-rowed, due to a too low MAF in the combined population. Missing data were imputed by the mean of each marker using the RR-BLUP package in R 4.2.2 (Endelman 2011). The imputation was carried out with the combined population for predictions within the combined population, and within each row-type and breeding set separately, for predictions with separate populations. The VanRaden kinship matrix was conducted and visualized using the GAPIT 3.0 package in R 4.2.2 (Wang and Zhang 2021) and principal component analysis (PCA) was conducted using the GAPIT 3.0 package in R 4.2.2 (Wang and Zhang 2021) and visualized using the plotly package in R 4.2.2 (Sievert 2020).

### Genomic prediction

Genomic prediction was carried out using the ridge regression best linear unbiased prediction (RR-BLUP) model (Endelman 2011) in R 4.2.2. The RR-BLUP model used was:

5 $$y = WGu + \varepsilon$$

where *W* is the design matrix relating lines to observations, *G* is the genotype matrix, *u* is a vector of marker effects and *ε* is the residuals. The predictive ability was estimated with fivefold cross-validation using 80% of the training population to predict the remaining 20% set as the validation population. The model was iterated with a randomized training population 500 times. The average accuracy of the predictions was determined by correlating the predicted values against the observed values of the validation population and average this correlation over the iterations. For predictions for single environments the BLUPs were used to predict the adjusted mean yield values which were used as the validation, i.e., a single year and location prediction.

## Results

### Realized genetic gain in market varieties

**Fig. 1:**
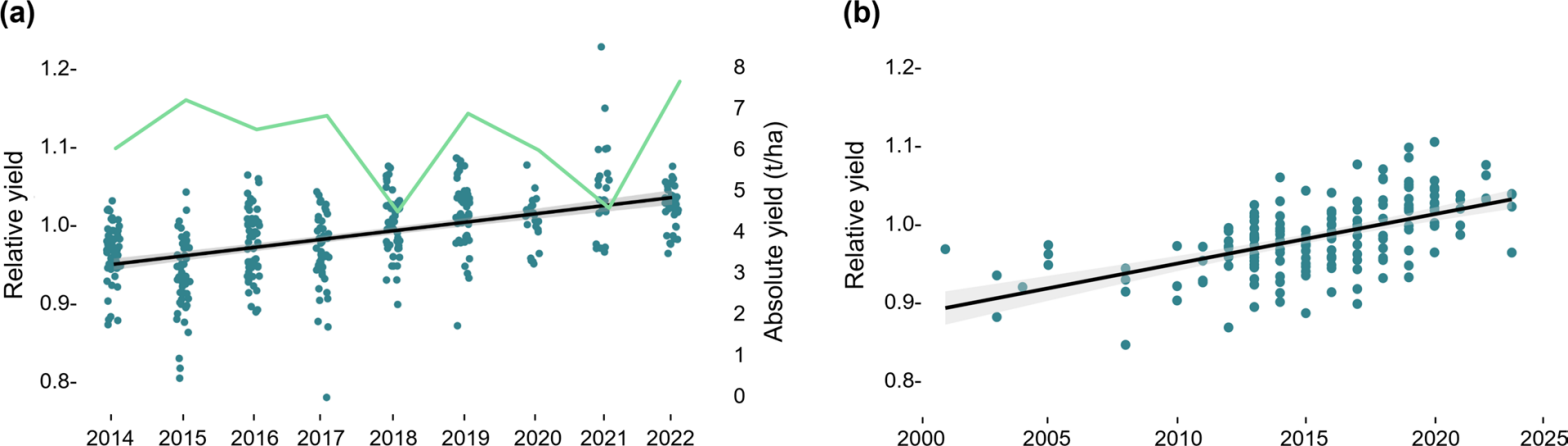
Relative and absolute yield of Swedish barley market varieties. **a** The genetic gain in a population of common market varieties tested in individual years increased by 1.07% from 2014 to 2022. The green line shows the absolute yield in tons/ha for the lines tested in each year as an average of the locations. **b** The genetic gain in varieties depending on year of release was 0.63% per year for market varieties registered 2001–2023

The genetic gain in spring barley market varieties (Table 2) was investigated as i) improvement of the population of market varieties available to growers, and ii) as improvement of registered market varieties. In the first scenario, the average increase in yield of the population of market varieties tested in individual years was 1.07% per year (Fig. 1a) suggesting that the population of varieties available to growers each year had improved yield compared to the synthetic tester. The increase in yield per year for the top five highest yielding varieties was 0.94%, whereas the lowest yielding varieties improved by 1.57% per year for the population of market varieties. In the second scenario, the average increase in yield of individual varieties compared to the synthetic tester was 0.63% per year for varieties registered 2001–2023 (Fig. 1b), suggesting that released varieties showed a genetic gain over this time.

**Table 2:** Number of lines and yield of spring barley varieties tested in official trials in 2014–2022

Year tested	Number of varieties	Relative yield	Absolute yield (tons/ha)
2014	60	0.96	6.03
2015	54	0.93	7.20
2016	55	0.98	6.49
2017	48	0.96	6.83
2018	50	1.01	4.49
2019	49	1.01	6.87
2020	22	1.00	5.98
2021	23	1.05	4.57
2022	37	1.02	7.65

Relative yield is the average yield of all varieties tested compared to the synthetic tester within each year. The absolute yield is the average yield for all varieties tested in each year

### Population structure and genetic variance in a barley breeding programe

**Fig. 2:**
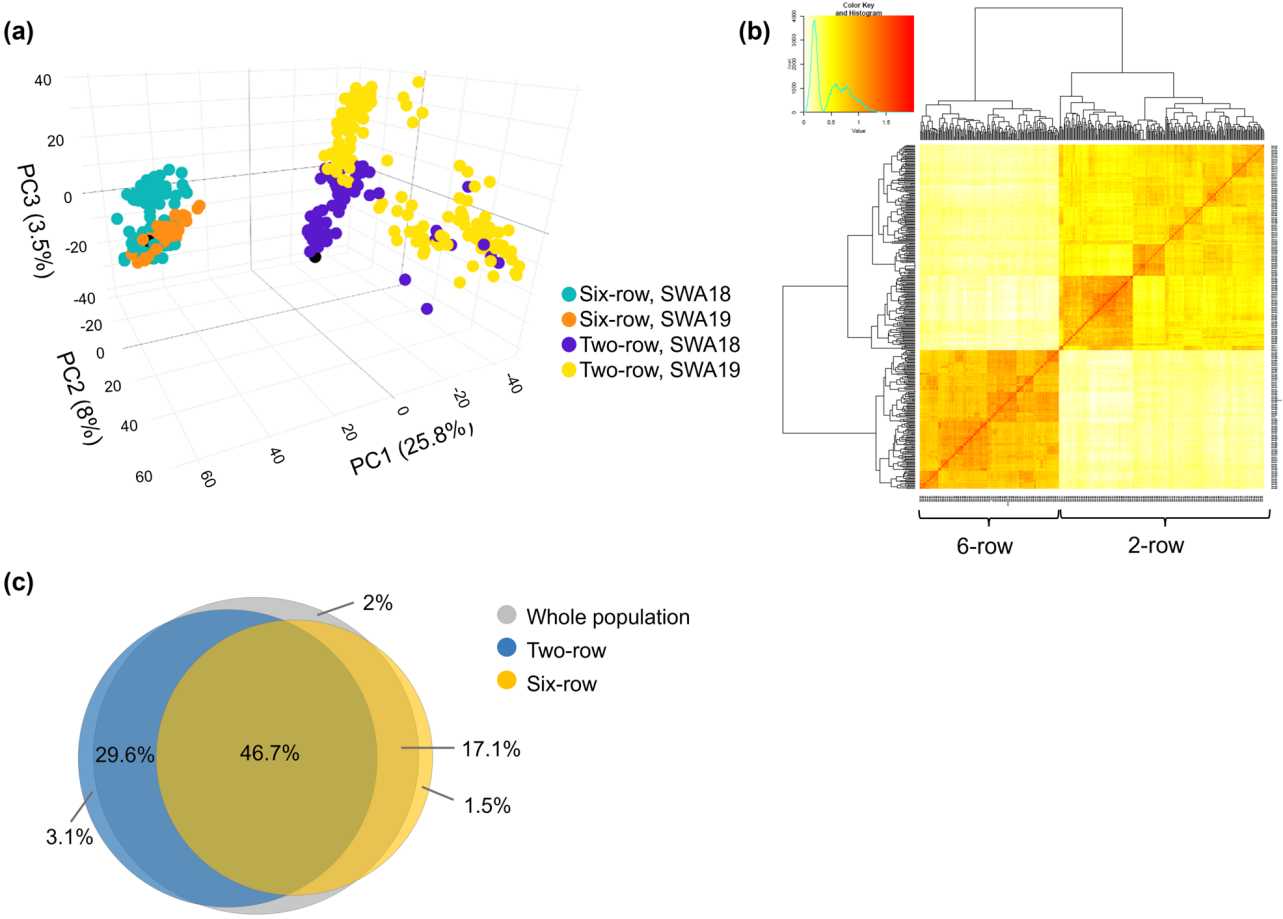
PCA plot, kinship heat map and markers with a MAF above 0.05 in the barley breeding lines. **a** Clustering of the genotypes depending on the row-type can be clearly identified from the PCA. The six-rowed genotypes clustered very tightly; however, within the two-rows, there was some diversity attributed to the breeding set in which the lines were developed. **b** Heat map of the VanRaden kinship matrix showing the relatedness of the lines depending on the row-type of the lines. The two-rowed lines display more clustering within the subpopulation compared to the six-rowed genotypes. **c** The proportion of markers with a MAF > 0.05 differed between the entire population and the groups of row-types, where 32.7% and 18.6% of the markers were polymorphic only in the two-row or the six-row lines, respectively

A principal component analysis (PCA) of the population detected the presence of two distinct subpopulations in the breeding population (Fig. 2a). The first principal component (PC) accounted for 25.8% of the genetic variation and separated the population depending on row-type, whereas the second PC accounted for only 8% variance. Within the two-row lines, there is some separation depending on the breeding set where the SWA19 appears more genetically diverse in the two-row lines. However, there is no such clear division within the six-row lines. This was also seen in the heat map of the kinship matrix where the lines were clearly separated depending on the row-type and some clustering was observed within the two-row genotypes (Fig. 2b). Although the difference between the breeding sets were only minor they were of interest for the possible effect on accuracy when conducting predictions between-breeding sets.

To analyze the difference in marker polymorphism in the breeding population the minor allele frequency (MAF) within the two-rowed and the six-rowed groups was calculated separately. The number of polymorphic markers, i.e., with a MAF above 0.05, differed depending on the row-type where 32.7% and 18.6% of the markers were uniquely polymorphic for either the two-row or the six-row lines, respectively, suggesting a significant difference in allele diversity between the row-types (Fig. 2c). Of the markers in the whole population, 2% were polymorphic only in the combined population, due to the fixation of different alleles in the separate row-type groups, i.e., non-informative markers for either row-type. Overall, only 46.7% of the markers were polymorphic in both row-type groups, suggesting that more than half of the markers were non-informative for the combined population.

### Grain yield is influenced by the interplay of row-type and environment

**Fig. 3:**
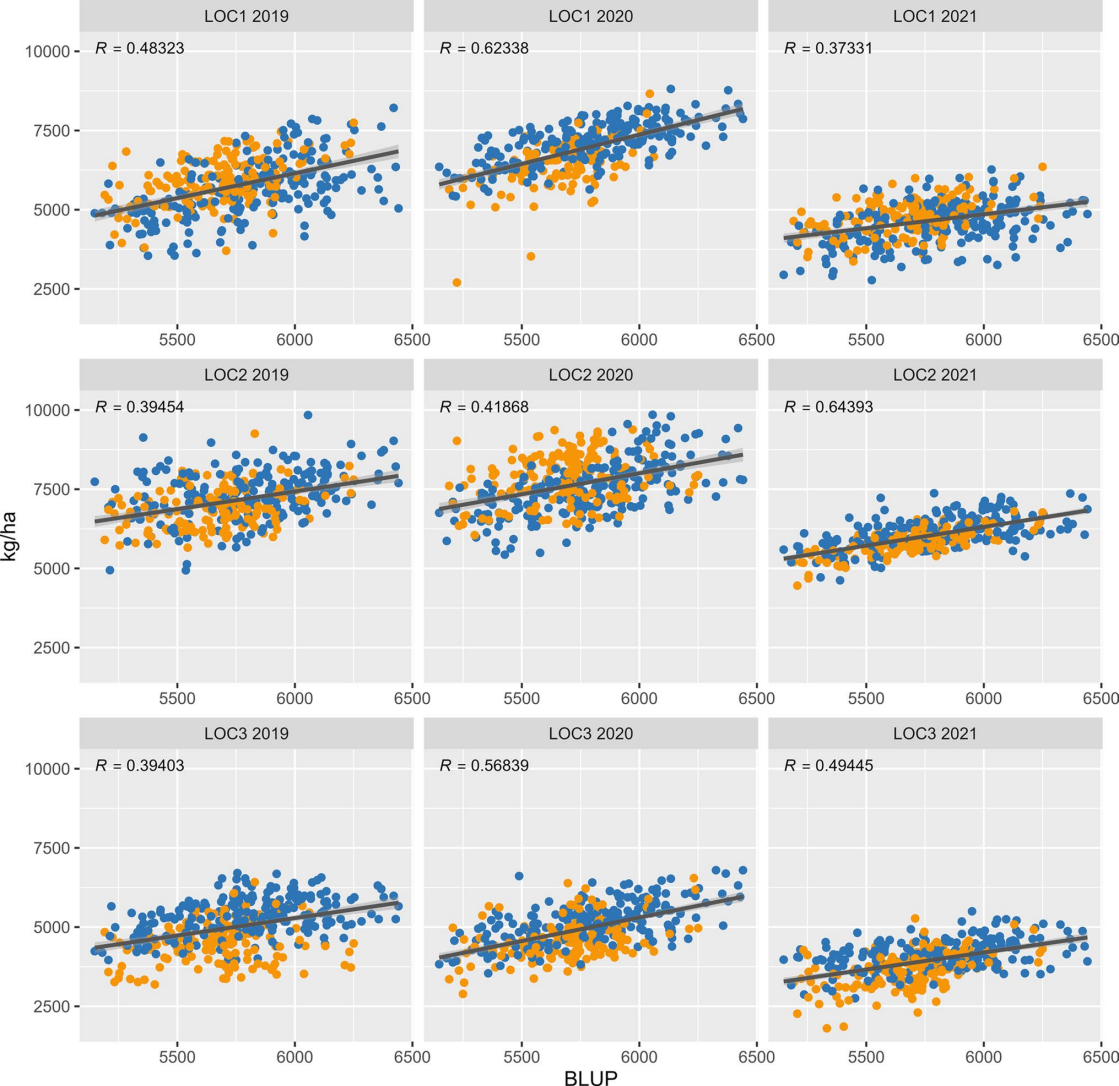
Correlation of the yield (kg/ha) in individual environments compared to BLUP values. All individual environments showed a medium to high correlation to the BLUP values as displayed with the Pearsons correlation coefficient (R) for each environment. The two-row lines (blue) were generally higher yielding compared to the six-row lines (orange) (colour figure online)

The broad sense heritability for yield (kg/ha) in the whole population was high (0.73) with a genetic variance of 0.089 (Table 3). The overall yield was higher in the two-rowed varieties than in the six-rowed, (p-value 9.4 × 10^–6^) with a higher genetic variance in the two-row lines. The two-rowed lines also showed a wider distribution of yield compared to the six-rowed, suggesting more diversity in the population (Table 3). In the individual environments 2021 was the lowest yielding year in all locations, whereas 2019 and 2020 were more comparable (Fig. 3), similar to the results from the genetic gain analysis (Fig. 1a). Location 2 was the highest yielding location, whereas the southernmost Location 3 was the lowest yielding location overall. The two-row varieties were generally higher yielding than the six-rowed varieties in the northernmost Location 1 and the reverse was true in the southernmost Location 3 (Fig. 3), suggesting that the row-type in this population might be of importance for the yield at different latitudes and climates.

**Table 3:** Broad sense heritability, genetic variance components, and population averages for yield (kg/ha) using BLUP values in spring barley

	All	Two-row	Six-row
Heritability (H^2^)	0.727	0.791	0.597
Genetic variance	0.089	0.104	0.056
Gen:year variance	0.011	0.003	0.026
Gen:loc variance	0.010	0.013	0.008
Year	0.705	0.720	0.648
Location	1.311	1.031	1.621
Year:location	0.107	0.235	0.025
Residual variance	0.239	0.197	0.239
Average (kg/ha)	5.74	5.95	5.51
Min (kg/ha)	4.47	4.97	4.47
Max (kg/ha)	7.13	7.13	6.55

### Genomic selection of targeted groups

The predictive ability (PA) for yield (kg/ha) in the entire population was 0.609 (Table 4, Scenario A). Due to the genetic difference between row-types, the breeding lines were divided depending on row-type to improve the PA. Interestingly, the PA decreased slightly to 0.572 within the two-row population and to 0.193 for the six-row lines (Table 4, Scenario B). Using markers which were only polymorphic within one row-type did not affect the PA, resulting in 0.579 and 0.189 for two-row and six-row lines, respectively. Using one row-type to predict the other (Table 4, Scenario C) resulted in low PA for both populations, suggesting that between row-type predictions are suboptimal.

**Table 4:** Predictive ability of genomic prediction of different training and validation populations for yield (kg/ha) in spring barley

Scenario	TP	VP	Pop size	TP size	VP size	PA yield
A. Whole population	All (80%)	All (20%)	375	300	75	0.609
B. Within row-type	Two-row	Two-row	222	178	44	0.572
	Six-row	Six-row	153	122	31	0.193
C. Between row-type	Two-row	Six-row	222	178	44	0.102
	Six-row	Two-row	153	122	31	0.210
D. Within-breeding set	SWA18	SWA18	178	142	36	0.437
	SWA19	SWA19	195	156	39	0.526
E. Between-breeding set	SWA18	SWA19	178	178	195	0.370
	SWA19	SWA18	195	195	178	0.326
F. Between years	2020 + 2021	2019	375	300	75	0.389
	2019 + 2021	2020	375	300	75	0.334
	2019 + 2020	2021	375	300	75	0.260
G. Between locations	Loc 1 + Loc 2	Loc 3	375	300	75	0.365
	Loc 1 + Loc 3	Loc 2	375	300	75	0.279
	Loc 2 + Loc 3	Loc 1	375	300	75	0.309
H. Within locations	Loc 1	Loc 1	375	300	75	0.357
	Loc 2	Loc 2	375	300	75	0.307
	Loc 3	Loc 3	375	300	75	0.487
I. Single location	All-Loc 1, 2019	Loc 1 in 2019	375	300	75	0.326
	All-Loc 1, 2020	Loc 1 in 2020	375	300	75	0.441
	All-Loc 1, 2021	Loc 1 in 2021	375	300	75	0.069

Yield data for 375 spring barley breeding lines tested in three locations and three years was used to test PA in different scenarios of training (TP) and validation (VP) population composition using fivefold cross-validation in RR-BLUP. PA,  predictive ability

The predictive ability within-breeding sets was slightly higher in the SWA19 set (0.527) than for the SWA18 set (0.437) (Table 4, Scenario D). The SWA19 generation had a higher proportion of two-rowed varieties which could explain why the PA was higher within this generation compared to the SWA18 which had a higher proportion of six-rowed lines. Using one breeding set to predict the GEBVs of another generation lead to a decrease in PA to 0.37 for SWA18 to SWA19 and 0.326 for SWA19 to SWA18 (Table 4, Scenario E), suggesting that between-breeding set predictions result in lower PA than within-breeding sets but is still a viable option. In addition, the division of the population into breeding sets and row-types resulted in smaller population sizes used to evaluate the prediction models, which could also have contributed to the lowering of the PA.

Predictions between years and locations were also carried out to investigate if predicting performance in an untested environment could be successful. The highest PA between different years was achieved by using data from 2020 and 2021, for lines in 2019, with a PA of 0.389 (Table 4, Scenario F). Of the years tested, the yield was the most stable across locations in 2019 which might have contributed to the higher PA. Interestingly, when using data from two locations to predict the third location, the PA was the lowest for the location with the overall highest yields, i.e., Location 2 (0.279), and highest for the location with the lowest overall yields, Location 3 (0.365) (Table 4, Scenario G).

Considering the different performance of the row-types depending on the location (Fig. 3), single location predictions were carried out to determine if the accuracy could be increased within a location, resulting in a PA of 0.357, 0.307, and 0.487 within locations 1, 2 and 3, respectively (Table 4, Scenario H). Again, the highest PA was achieved for the lowest yielding Location 3 and the lowest PA for the highest yielding location 2.

A more realistic scenario for a breeding programe that targets a specific region, for example Location 1, would be to test some material in the target region and investigate whether adding data from other regions would improve the PA of untested lines. In this approach, the PA varied significantly depending on the year within Location 1 (Table 4, Scenario I). Using 2019 and 2021 to predict the yield in 2020, which was the highest yielding year, resulted in the highest PA (0.441), whereas using data from 2019 and 2020 for the prediction of 2021, the lowest yielding year, resulted in the lowest PA (0.069). There was more variability in the PA when predicting only one environment compared to predicting one location with three years of data. This follows naturally from the increase in variability in the line performance in each environment.

## Discussion

### Genetic gain

The continued yield gain increasingly depends on progress from plant breeding (Lillemo et al. 2010; Mackay et al. 2011). Understanding the historical and current gains achieved through breeding is essential to set future targets and evaluate the performance of new breeding strategies. Here, field trial data were collected to compare varieties commonly grown in Sweden, i.e., KWS Irina, Dragoon and RGT Planet, to a population of current and candidate market varieties. This approach made it possible to investigate the genetic gain of material currently available to farmers. Within this population of varieties marketed for this region, there was a trend of genetic gain increase for the yield of 1.07% per year, compared to the checks, suggesting that there is progress in the breeding for better-performing lines. The yield of the top performers in each year showed a smaller improvement of 0.95% per year, whereas the lowest yielding lines improved by 1.57% per year. It is unclear whether this improvement of the worst-performing lines was due to improvement or to the removal of lines included in the field trials. However, new lines were continuously added to the population over the tested years, and they performed better than the lines removed from the trials with an average of 0.63% genetic gain in line performance per year.

Ortiz et al. (2002) studied genetic gain in Nordic spring barley and found a genetic gain of 13% over 60 years of breeding. Finnish spring barley varieties showed no genetic gain in varieties released from 1927 to 1980 (Peltonen-Sainio and Karjalainen 1991). The lines used in the current study were all registered on the Swedish market from 2001 to 2023 and constitute an estimate of the genetic gain in the varieties available to farmers. The overall gain of the varieties in this study of 1.07% per year is one of the highest reported for spring barley, suggesting a steady increase in yield potential over time; however, there is a need to improve the genetic gain to meet future demands.

Increasing genetic gain in future market varieties remains a challenge but also provides opportunities for new technology. Through simulated trials with varying population sizes and trait heritability, the genomic selection strategy constantly outperformed phenotypic or marker-assisted selection (MAS) in predicted genetic gain over time (Bernardo and Yu 2007; Iwata and Jannink 2011; Jannink 2010). In barley breeding, GP using RR-BLUP resulted in a higher genetic gain increase in comparison with traditional phenotypic selection which was especially true for traits with low heritability and for populations with large sample sizes (Iwata and Jannink 2011). By using GP, the expected genetic gain per year can be two to three times higher for wheat and maize in comparison with the use of MAS or phenotypic selection (Heffner et al. 2010; Tessema et al. 2020). Integrating GP in breeding programs can potentially increase genetic gain while reducing cost per unit time (Heffner et al. 2010; Lorenz 2013).

### Genomic selection for targeted regions

For growers, the selection of varieties for cultivation could be assisted by variety testing in locations resembling their own conditions (Cooper et al. 1996; Smith et al. 2015). However, for breeding, the prediction of the performance of lines in larger target regions still lacks in the predictive ability of new environments (Smith et al. 2015). In this study, using observed environments to predict untested environments (the combination of a year and a location) led to moderate predictive ability (Table 4). The variation between trial sites within a region can be as large as the variation between regions suggesting that non-static genotype by environment interactions due to seasonal factors can be higher than regional differences (Cullis et al. 2006).

A higher PA depends on designing the right training set from a carefully designed breeding scheme (Bassi et al. 2016). Training population size is one of the most important considerations for the PA in GS (Asoro et al. 2011; Isidro et al. 2015; Lorenzana and Bernardo 2009), more so than the marker density and number, as it affects the diversity and the genetic variance that can be identified in a crop. The accuracy of predictions is usually decreased if there is little relatedness between the training and the breeding population (Gianola et al. 2009; Habier et al. 2007, 2010; Zhong et al. 2009). In wheat, the PA for a validation population of little relatedness to the training population could be improved by increasing the diversity of the training set (Norman et al. 2018). In contrast, using populations with low relatedness decreased the PA from an overall of 0.8–0.4 (Norman et al. 2018).

The population structure caused by the row-types in spring barley is of importance for the implementation of GP methods in breeding programs. Although the genetics resulting in the fertility of the lateral spikes, and thereby the two- or six-rowed phenotype, is relatively simplistic and well-known, the extent of the difference in genetics resulting from the separation of the populations is less known. In a study with four wild barley (three *Hordeum ssp. spontaneum* and one *Hordeum spp. agriocrithon*) and a diversity panel of cultivated inbred barley lines, the most genetic variation was attributed to the row-type, rather than the cultivation status, and accounted for 15.1% of the genetic variation (Casale et al. 2022). Another study found differences in allele frequency between two- and six-rowed barley, potentially due to the incorporation and fixation of important loci related to yield, disease resistance, abiotic stress resistance, and flowering time (Wonneberger et al. 2023). Breeding of barley in modern times has focused on adapting barley for a variety of different purposes and environments. Higher protein content for varieties aimed for animal feed, and low protein and large grain size for malting barley varieties could have contributed to the differences in the germplasms. In addition, alleles for powdery mildew resistance (Friedt et al. 2011; Jørgensen 1992), selection for late flowering, and increased yield in European two-rowed spring barley (Tondelli et al. 2013) led to further separation between row-types.

The spring barley population used in the current study consisted of lines of different row-types and from different breeding sets. Here, there was significant genetic difference depending on row-type, accounting for 25.8% of the genetic variation, with less diversity assigned to breeding set. When all lines were used as one population the PA was high, 0.609, whereas predictions within the individual row-type groups achieved accuracies of 0.572 and 0.193 for two-row and six-row accessions, respectively. The genetic separation of the row-types in barley also resulted in very low PA when using one row-type to predict the other whereas the inter-breeding set prediction was still moderate (Table 4, Scenario C and E). This could indicate that the high PA for the whole population was inflated, due to the clustering of the genetic subpopulations depending on row-types. Predictions using whole populations can lead to higher PAs compared to within-subpopulation predictions (Guo et al. 2014; Werner et al. 2020) suggesting that the accounting for population structure is important for accurate prediction estimates.

Another factor influencing GP accuracy is the size of the training population. When separating the population based on row-types or breeding sets there was a significant reduction in effective population size for each training population which could have contributed to the lowering of the PA compared to the use of the entire population. In a study on barley, Nielsen et al. (2016) found that reducing the training set to less than 200 lines decreased the PA. In addition, with a lower training population the importance of the population structure and the relatedness of the lines is increased (Nielsen et al. 2016). To account for population structure such as the row-type in barley through the division of the training population, the overall size of the training population decreases, resulting in a reduced PA. Including more observations could be necessary to improve the predictions to accurately account for the differences between the of the spring barley row-types.

## Conclusion

Genetic gain per year in current market varieties was estimated as 1.07% for the varieties tested. This estimate can serve as a checkpoint for the evaluation of new varieties currently developed. Introducing genomic prediction in plant breeding programs has the potential to streamline selection in breeding programs. In barley separating the population based on row-type to increase the predictive ability in genomic selection could be beneficial if the number of lines in the breeding population is large enough. Large populations might be needed to account for population differences depending on row-type to improve the predictive ability of a divided population, whereas programs with a low number of one or both row-types might benefit from keeping the population intact. Targeted breeding for untested regions could significantly reduce phenotyping costs; however, the effects of the variations in the environments, i.e., years and locations, somewhat reduced the predictive ability.

## Supplementary Information

Below is the link to the electronic supplementary material.

Supplementary file1 Accessions included in the genetic gain analysis. The year of release, the breeding company registered with the variety is specified along with the first year and the number of times it was used in the trials.(XLSX 18 KB)

## Data Availability

The datasets analyzed for genetic gain estimation during the current study are available at the Sverigeförsöken repository (https://sverigeforsoken.se/).
